# High-Resolution Melting PCR as a Fast and Simple Molecular Biology-Based Method for the Identification of Hypervirulent *Clostridioides difficile* Strains Directly in Stool Samples

**DOI:** 10.3390/microorganisms12112228

**Published:** 2024-11-03

**Authors:** Tomasz Bogiel, Robert Górniak, Weronika Ambroziak, Szymon Zieliński, Dominika Anna Zieja, Piotr Kanarek

**Affiliations:** 1 Microbiology Department Ludwik Rydygier, Collegium Medicum in Bydgoszcz, Nicolaus Copernicus University in Toruń, 9 Maria Skłodowska-Curie Street, 85-094 Bydgoszcz, Poland; 2 Microbiology Student Science Club, Ludwik Rydygier Collegium Medicum in Bydgoszcz, Nicolaus Copernicus University in Toruń, 9 Maria Skłodowska-Curie Street, 85-094 Bydgoszcz, Poland; robert.gorniak99@gmail.com; 3 Clinical Microbiology Laboratory, Dr. Antoni Jurasz University Hospital No. 1 in Bydgoszcz, 9 Maria Skłodowska-Curie Street, 85-094 Bydgoszcz, Poland; werr25@wp.pl; 4 Laboratory of Genetics and Molecular Biology, Prof. Dr. Stanisław Popowski Regional Specialized Children’s Hospital in Olsztyn, 18a Żołnierska Street, 10-561 Olsztyn, Poland; dominika.anna.gruchala@gmail.com; 5 Medical Laboratories Bruss Alab Group Sp. z o.o., 9B Powstania Styczniowego Street, 81-519 Gdynia, Poland; szymenzi@gmail.com; 6 Department of Microbiology and Food Technology, Faculty of Agriculture and Biotechnology, Bydgoszcz University of Science and Technology, 85-029 Bydgoszcz, Poland; piokan004@pbs.edu.pl

**Keywords:** binary toxin, *Clostridioides difficile*, diarrhea, high-resolution melting, real-time HRM-PCR

## Abstract

*Clostridioides difficile* became one of the main causes of nosocomial infections in all clinical settings worldwide, especially among patients undergoing antibiotic therapy. The incidence and severity of *C. difficile* infections, from mild diarrhea to life-threatening pseudomembranous colitis, correlate with the spread of the hypervirulent binary toxin (CDT)-producing strains. The use of the real-time HRM-PCR method enables the identification of hypervirulent *C. difficile* strains directly in the diarrheal stool samples of patients suspected of being infected with this bacterium. For this purpose, the *cdtA* and *cdtB* genes encoding CDT subunits, as well as the species-specific *gluD* gene, were detected to identify the presence of this bacterium in the tested samples. The sensitivity, specificity, negative predictive value (NPV) and positive predictive value (PPV) of the established method were also assessed. The obtained results were compared with the results of eazyplex^®^ C. difficile complete test (AmplexDiagnostics GmbH) based on the LAMP method, used in standard microbiological diagnostics. The values of the assessed diagnostic parameters for the detected genes ranged from 58.82% to 98.85%. The lowest value (58.82%) was obtained for the PPV of *cdtB* and the highest (98.85%) for the NPV of this gene. The real-time HRM-PCR method enables fast and simple detection of the investigated genes of hypervirulent *C. difficile* strains and, after careful optimization, may demonstrate high potential for usefulness in routine microbiological diagnostics.

## 1. Introduction

At the end of the 20th century, there was a significant increase in the frequency of *Clostridioides difficile* infections (CDIs), with them becoming one of the main causes of nosocomial outbreaks in a number of clinical settings, especially among patients undergoing antibiotic therapy [1]. The clinical symptoms of these infections range from mild diarrhea to pseudomembranous colitis, which is a life-threatening condition [2]. The incidence and severity of infections with *C. difficile* strains correlate with the appearance of the hypervirulent CDT-producing strains, mostly BI/NAP1/027 and especially its endemic spread. Epidemics caused by this strain have become and still are the main global cause of antibiotic-associated diarrhea in health care facilities worldwide [3].

CDT (*Clostridioides difficile* binary toxin) consists of two unconnected molecules—binary toxin subunit A (CDTa) and binary toxin subunit B (CDTb). CDTa, with a molecular mass of 48 kDa, is encoded by a 1392 base pair (bp) sequence and acts as an enzymatic component, conferring the activity of adenosine-5′-diphosphate ribosyltransferase. In turn, CDTb with a molecular mass of 94 kDa, encoded by a gene consisting of 2631 nucleotides, is a binding component, enabling the passage of the enzymatic subunit into the human cell cytosol [4].

A functional CDT is encoded by a synergistic expression of the products of the binary toxin subunit A gene (*cdtA*) and the binary toxin subunit B gene (*cdtB*), both located in a region called the “CDT locus”, with a size of 6.2 kilo bp. This region also contains the *cdtR* gene, which is involved in the positive regulation of CDT synthesis and the production of both TcdA and TcdB. The lack of a functional *cdtR* gene causes an approximately 15-fold decrease in CDT synthesis level. This regulation occurs at the transcriptional level, probably through an indirect control of the TcdR-positive regulator of PaLoc region expression [5,6].

As a result of proteolytic cleavage, CDTb is activated and binds to the cell surface of the exposed CDT target, the lipolysis-stimulated lipoprotein receptor (LRS), forming heptamers. CDTa is then attached, and the toxin–receptor complex is endocytosed. Due to the low pH of the endosome, a conformational change in the formed heptamers occurs. This leads to the formation of a membrane with the formation of pores through which CDTa enters the host’s cell cytosol. This process depends on host chaperones, including heat-shock proteins and cyclophilin A. Once it infiltrates inside the cell, the enzymatic component ribosylates monomeric adenosine diphosphate G-actin, thereby inhibiting the polymerization of G-actin to F-actin. As a result, the depolymerization of the actin cytoskeleton is enhanced, driven by toxin-dependent induction of new microtubule structures. They consist of long, microtubule-dependent protrusions on the surface of epithelial cells that increase the surface area for interaction and therefore promote attachment and further colonization of *C. difficile* [3,7,8].

PCR is commonly known for its high specificity and sensitivity. However, the PCR product should always be checked for amplification specificity. This step is highly accurate while using electrophoretic separation of amplicons and size confirmation of the PCR product under UV visualization. However, this becomes more challenging with real-time PCR based on intercalating dyes. Therefore, controlling the specificity of the real-time PCR technique can be achieved through the high-resolution melting (HRM) method of the amplified sequence. HRM is based on the identification of differences in the shape of melting curves generated by temperature changes during the denaturation of the PCR products in the presence of fluorescent dyes such as SYBR Green or EvaGreen. These dyes bind to single- or double-stranded DNA chains by intercalation between adjacent bps and emit a signal when excited with light of a specific wavelength. They thus enable the identification of small changes in nucleic acid sequences by generating DNA melting curve profiles of PCR amplicons. This enables the determination of the specificity of the amplification reaction by confirming the presence of appropriate PCR products corresponding with the applied controls, and excluding non-specific PCR products from the analysis [9,10].

In routine CDI diagnostics, the eazyplex^®^ *C. difficile* complete test (AmplexDiagnostics GmbH, Gars am Inn, Germany) is commonly used. It is based on loop-mediated isothermal amplification (LAMP) and is one of the relatively few kits to also enable binary toxin gene identification. LAMP is a highly specific amplification technique resulting from the use of four to six primers. Its application increases the number of amplified DNA fragments up to one billion copies in less than an hour. The sensitivity and specificity of LAMP are comparable to those of PCR, but the results can be obtained in a shorter time [11]. The reaction based on the LAMP technique takes place in a heating block or water bath at a constant temperature, usually 60–65 °C. This eliminates the need for use of a thermal cycler as laboratory equipment. Amplification is possible due to the use of DNA polymerase with the activity of what is known as thread replacement. When the investigated DNA in the sample is complementary to the amplification primers, a series of DNA loops are created. With each subsequent reaction cycle, the loops become more complex and numerous. Detection of the obtained products is possible thanks to the use of either fluorescent dyes, ultraviolet irradiation, agarose gel electrophoresis, turbidity changes or real-time fluorescence intensity read-outs [12,13].

The aim of this study was to expand the current state of knowledge of binary toxin-producing *C. difficile* strains, especially in the field of diagnostics related to the incidence of infections with toxinogenic *C. difficile*, binary toxin-producing isolates, and to establish and determine the usefulness of the real-time HRM-PCR method for the detection of hypervirulent strains directly in stool samples.

## 2. Materials and Methods

### 2.1. The Origin of Clinical Material for Research

The tested material consisted of 276 samples (DNA lysates) derived from stool samples remaining after the diagnostic process for the presence of *C. difficile* infections. All the samples were collected for routine testing in 2021–2023 at the Department of Clinical Microbiology from patients hospitalized at the Dr. Antoni Jurasz University Hospital No. 1. in Bydgoszcz, Poland. The diarrhea stool samples used were derived from different patients from different hospital units (only one sample per patient).

Moreover, in order to validate the obtained results, two samples from the international quality assessment program in laboratory medicine (Labquality) were used.

### 2.2. Preparation and Storage of Clinical Material for Research Purposes

The tested samples were prepared by introducing 10 μg of diarrheal feces into 500 μL of LPTV buffer (AmplexDiagnostics GmbH, Gars am Inn, Germany). Then, the mixed samples were incubated for three minutes at 99 °C and centrifuged for 60 s at 3900 rpm (MiniSpin plus, Eppendorf, Germany). The obtained lysates were labeled and used for diagnostic purposes. The residue aliquots were frozen at −20 °C and, after thawing, used for the current research.

### 2.3. Detection of Clostridioides difficile Genes Encoding a Binary Toxin and the Species-Specific gluD Gene

In order to detect genes encoding *C. difficile* CDT subunits, the real-time HRM-PCR technique was used. In addition, to identify the presence of bacteria of this species in the tested samples, the experiment was enriched with the detection of the species-specific glutamate dehydrogenase gene (*gluD*). Therefore, the following genes were detected in separate reactions in the tested samples: *cdtA*, *cdtB* and *gluD* (Table 1). Their presence was determined using the following devices: CFX Opus 96 Dx Real-Time PCR System (BioRad, Hercules, CA, USA) and LightCycler^®^ 480 II (Roche, Rotkreuz, Switzerland), with the *cdtB* gene detected using the CFX Opus 96 Dx Real-Time PCR System analyzer only. All the reactions were performed in separate wells of 96-well optical reaction plates with a volume of 200 μL (Roche, Rotkreuz, Switzerland). Positive and negative controls were also used for each batch of assays. These were, respectively, genomic DNA isolated from the CDT-producing *C. difficile* strain ribotype 027, serving as reference strain for all of the investigated genes, and water for molecular biology purposes (Eppendorf, Hamburg, Germany). The thawed and mixed components of the reaction mixture were pipetted under the sterile conditions of a laminar chamber into a sterile Eppendorf tube, the contents of which were mixed again. The composition of the reaction mixture was as follows: ready-to-use 5× concentrated HOT FIREPol^®^ EvaGreen^®^ HRM Mix solution (SolisBiodyne, Tartu, Estonia) containing the EvaGreen^®^ dye and all components necessary to perform the real-time HRM-PCR reaction (HOT FIREPol^®^ DNA polymerase, ultrapure dNTPs and MgCl_2_), molecular biology grade water and the corresponding primers (Table 1). A summary of the concentrations and volumes of the reagents used is presented in Table 2, and the detailed specifications of the primers used are presented in Table 1 (one pair of forward and reverse primers each for *cdtB* and *gluD* and two forward with one reverse primer for *cdtA*, according to European Centre for Disease Prevention and Control Recommendations, https://www.ecdc.europa.eu/en/clostridioides-difficile-infections) URL (accessed on 20 September 2024). Seventeen µL of the reaction mixture was pipetted into a dedicated well of the reaction plate and 3 µL of the tested sample or DNA of the standard strain (positive) or negative control was added, then the well was carefully sealed with adhesive foil (BRAND GMBH + Co KG, Wertheim, Germany) and placed in the thermocycler for amplification. The concentrations of reagents and selection of the wavelength range, analogous to that for the SYBR Green dye, were set up according to the HRM Mix manufacturer’s instructions.

The amplification conditions of the real-time PCR performed in both thermal cyclers were the same, set according to the HRM Mix manufacturer’s instructions, with initial denaturation at 95 °C for 12 min, followed by 50 cycles consisting of: denaturation at 95 °C for 15 s, primer annealing at 65 °C (checked initially and optimized for the purposes of this study) for 20 s and primer extension at 72 °C for 20 s. The analysis of the melting curve of the amplification product was performed using a temperature increase from 65 °C to 97 °C with increments of 0.5 °C every 5 s and constant read-outs. The entire reaction took about two hours.

**Figure 1:**
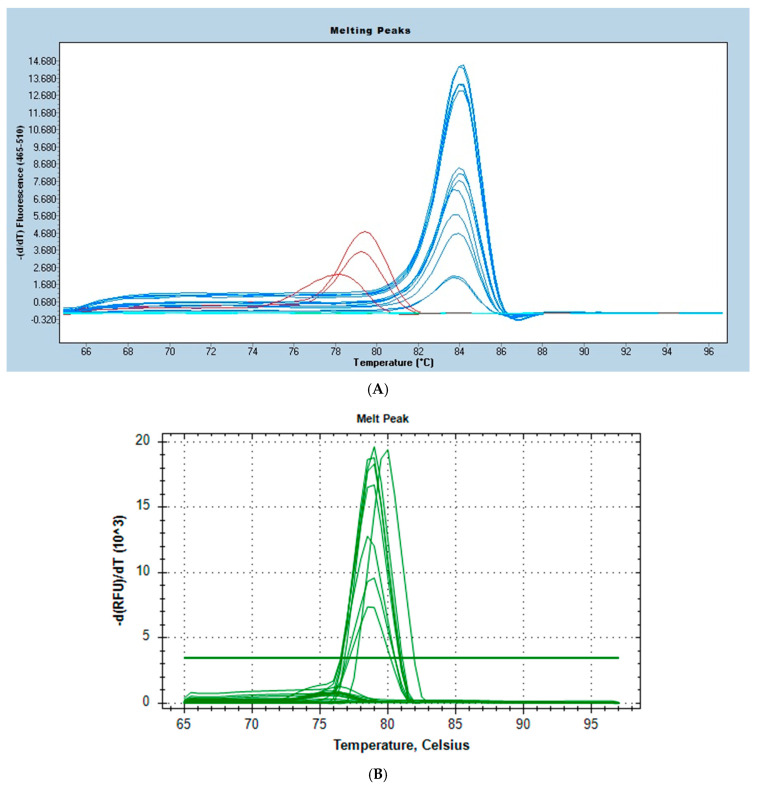
The examples of logarithmized melting curve fluorescence intensity values of the: (**A**) *gluD* gene amplification product obtained by real-time HRM-PCR using the LightCycler^®^ 480 II apparatus (Roche, Rotkreuz, Switzerland) where *gluD*—lines showing fluorescence intensity obtained for glutamate dehydrogenase gene amplification; blue curves—melting charts of the specific *gluD* gene amplification product (positive results); red curves—melting of the unspecific amplification product (negative results); and (**B**) the examples of melting curves of the *cdtB* gene amplification product obtained by the real-time HRM-PCR method using the CFX Opus 96 Dx Real-Time PCR System thermal cycler where *cdtB*—binary toxin subunit B gene; green curves above the baseline—melting curves of the specific product of *cdtB* gene amplification (positive results); green curves below the baseline—melting curves of the non-specific amplification product (negative results).

During the first stage of the reaction, HOT FIREPol^®^ DNA polymerase was activated by a 12 min incubation at 95 °C. This prevents the amplification of sequences with non-specifically attached primers and the formation and subsequent amplification of primers–dimers at lower temperatures during the assay. The specificity of the final amplification products was assessed based on the fluorescence intensities of the obtained melting curves and their automatic logarithmized calculations and visualizations. Table 3 shows the melting temperature (Tm) peak values of the determined genes. The visual examples of the results of the specificity evaluation for both thermal cyclers used are shown in Figure 1. Amplification peaks falling within the temperature range of the reference strain genes were classified as positive, while those showing different shapes and melting temperature peaks were considered non-specific products. Their appearance was equivalent to the lack of an appropriate complementary amplification product. Therefore, such results were classified as negative.

### 2.4. Diagnostic Parameters of the Applied Methodology

The usefulness of the real-time HRM-PCR method for detecting CDT subunit genes and the *gluD* gene was assessed based on the determination of diagnostic sensitivity and specificity as well as positive predictive value (PPV) and negative predictive value (NPV). The comparison was made against the results obtained using the IVD certified kit—the eazyplex^®^ *C. difficile* complete test (AmplexDiagnostics GmbH, Gars am Inn, Germany) using the LAMP technique. The results obtained by this method are used in standard hospital microbiological diagnostics. Comparison with archived results of individual samples was possible after checking the diagnostic results in the PROMic^®^ IT (version 2024, Ostaszewo, Poland) system serving the microbiology laboratory.

A false-negative (FN) result was defined as the failure to detect the presence of *gluD* or one of the genes encoding CDT by real-time HRM-PCR, while the gene encoding GDH or *cdt* were detected by the LAMP method using eazyplex^®^ tests (AmplexDiagnostics GmbH, Gars am Inn, Germany). In turn, a false-positive (FP) result was considered to be the detection of the gene with the real-time HRM-PCR method, but not its detection using the eazyplex^®^ kit. (AmplexDiagnostics GmbH, Gars am Inn, Germany) FP results also included ambiguous results—when the presence of only one gene encoding the CDT subunit was detected when the *cdt* gene was detected using eazyplex^®^ tests. Positive results for the two methods were considered true positives (TPs). A similar situation occurred for the classification of true-negative (TN) result consistency, negative in both methods.

In order to minimize the occurrence of FN and FP results, any differences in the results obtained with respect to the eazyplex^®^ tests were verified by retesting the samples. For only one gene encoding CDT subunit detection, the testing for both genes was performed three times to minimize the risk of false results. If the determinations were not completely consistent, the result obtained twice was treated as the final result. Additionally, the retesting for TP and TN results was performed on several randomly selected samples to assess the repeatability of the method. Determination of the diagnostic parameters of the method was possible thanks to the use of the following commonly used mathematical formulas:

- $\mathrm{Test}\;\mathrm{sensitivity}\;=\frac{\mathrm{TP}}{\mathrm{TP}+\mathrm{FN}}\times 100\%$
- $\mathrm{Test}\;\mathrm{specificity}\;=\frac{\mathrm{TN}}{\mathrm{TN}+\mathrm{FP}}\times 100\%$
- $\mathrm{PPV}=\frac{\mathrm{TP}}{\mathrm{TP}+\mathrm{FP}}\times 100\%$
- $\mathrm{NPV}\;=\frac{\mathrm{TN}}{\mathrm{TN}+\mathrm{FN}}\times 100\%$

### 2.5. Statistical Analysis

Statistical analysis was performed using Excel (Microsoft, 20 September 2023) and STATISTICA 13.3 (StatSoft) software. The obtained results were verified using a significance coefficient of α = 0.05, which made it possible to recognize as statistically significant differences occurring with a test probability of *p* < 0.05.

In order to compare differences in the frequency of genes encoding CDT subunits and GDH in the examined group of samples, the classic chi-square (χ^2^) test of agreement was used.

Using Excel (Microsoft), the percentage frequency of *cdtA* and *cdtB* gene co-occurrence in clinical samples was also calculated. The repeatability of the method was assessed by dividing the total number of concordance results by the number of repeated determinations in the same sample in terms of the presence of a given gene. A compliance was understood as an obtaining a positive or negative result twice.

## 3. Results

A total of 276 DNA samples/lysates isolated from stool samples remaining after the diagnostic process were examined to assess the presence of the *cdtA*, *cdtB* and *gluD C. difficile* genes. The *gluD* gene was detected in 189 samples, including both samples from the international laboratory medicine quality assessment program Labquality. Moreover, in one of the Labquality samples, the presence of genes encoding CDT subunits was also found. Therefore, the results of external laboratory control samples obtained during the analysis were identical to those obtained in standard hospital microbiological diagnostics. They were additionally consistent with the assumptions of the quality control test, underlining the specificity and sensitivity of the established methodology.

### 3.1. Assessment of Differences in the Frequency of the Detected Clostridioides difficile Toxin Genes

The *cdtA* gene was noted in 15 (5.4%), while *cdtB* gene in 17 (6.2%) of the tested samples. Using STATISTICA 13.3 (StatSoft), the chi-square test was used to compare the level of statistical significance of differences in the frequency of detected *C. difficile* genes in clinical samples. A *p* value of 0.0277 was obtained, indicating the statistical significance of differences in the distribution of the number of genes. The obtained results are presented in Table 4.

**Figure 2:**
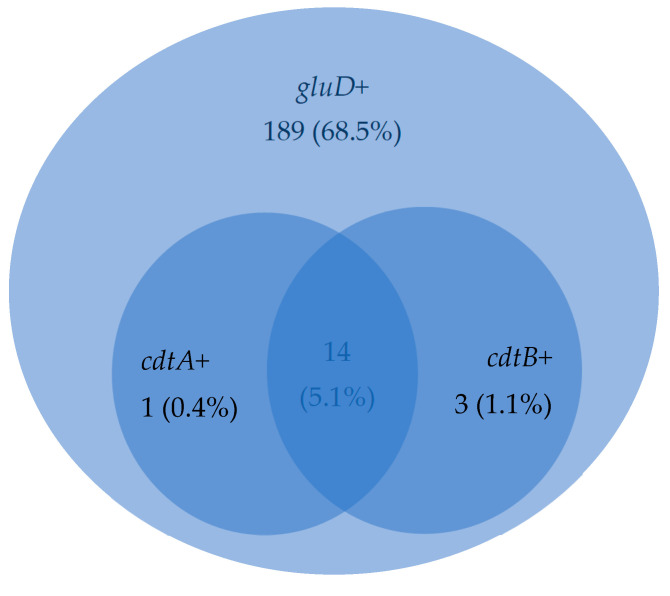
Diagram showing the frequency of co-occurrence of the *gluD*, *cdtA* and *cdtB* genes (*n* = 276), where *cdtA*+—the presence of binary toxin subunit A gene; *cdtB*+—the presence of binary toxin subunit B gene; gluD—the glutamate dehydrogenase gene; *n*—the number of samples with detected *gluD* and *cdtA* and/or *cdtB* gene.

The frequency of co-occurrence of the *gluD*, *cdtA* and *cdtB* genes for clinical samples in which these genes were detected was calculated as a percentage. The obtained results are presented in Figure 2.

### 3.2. Assessment of Diagnostic Parameters of the Applied Methodology

The results obtained using the real-time HRM-PCR method were compared with the eazyplex^®^ test results, including the two Labquality-derived samples. They are presented in Table 5. The results for the *gluD* gene were characterized by the greatest discrepancy. For all the detected genes, more of the obtained results were FP than FN.

Based on the obtained differences and agreement scores, the diagnostic sensitivity and specificity as well as the PPV and NPV were calculated. The obtained results are presented in Table 6. The values of the assessed diagnostic parameters for the tested genes ranged from 58.82% to 98.85%. The lowest value was obtained for the PPV of *cdtB* investigation and the highest for the NPV of this gene detection. The parameters of the diagnostic evaluation of CDT-encoding genes were similar. The highest detection sensitivity of 98.11% and the lowest specificity of 70.59% were obtained for the *gluD* gene.

### 3.3. Assessment of the Repeatability of the Applied Methodology

A repeated testing for the detection of the same gene in the same sample made it possible to determine the repeatability of the method used. The detection of the *cdtA* gene had the highest repeatability of 77.42%. The lowest detection rate was noted for the *gluD* gene. In this case, the repeatability was 64.52%. The obtained results are presented in Table 7.

## 4. Discussion

The main virulence factors of *C. difficile* are toxins A and B, abbreviated as TcdA and TcdB. However, when diagnosing infections, one should not omit the third important toxin—CDT, the role of which in CDI pathogenesis was largely ignored a few years ago. This binary toxin consists of two subunits—CDTa and CDTb, which are produced and secreted as two polypeptides, resulting in a potent toxin activity [14]. This toxin is produced by some strains, including the endemic hypervirulent strain BI/NAP1/027, associated with an increased frequency and severity of infections caused by *C. difficile* strains [4]. In this study, the incidence of CDT was assessed based on the detection of genes encoding subunits of this toxin using the real-time HRM-PCR technique. Moreover, the usefulness of the established method used for hypervirulent strains detection was also assessed.

According to the available literature, both domestic and foreign, there is a lot of data on the toxicity of *C. difficile*. There is also a focus on detecting the CDT toxin genes or the toxin itself. For this task, various methods and detection techniques have been used in the relevant literature. However, to the best of our knowledge, this work is the first example of the use of the real-time HRM-PCR technique for this purpose and especially for its application directly on stool samples, without the necessity of *C. difficile* strains being cultured.

A few published works discuss the use of real-time HRM-PCR, especially in the context of *C. difficile* detection or strains characteristics. The above-mentioned method was already used in 2012 by Grando et al. [15] to differentiate ribotype 027 of *C. difficile* from others of this species. The analysis was performed on 93 clinical isolates and five control strains. Ribotyping yielded unequivocal results for 88 of 98 isolates tested. These isolates belonged to 18 different ribotype groups with 70% homology. One of the clinical isolates showed 100% homology with the control strains of 027 ribotype. In the cited study, the application of real-time HRM-PCR successfully allowed to confirm the detected and control strains of this ribotype.

The real-time PCR technique, modified to detect many genes during one reaction, enabled the creation of an innovative test developed by Angione et al. [16], based on the analysis of the melting curve using a single fluorophore. This enables the identification and subtyping of *C*. *difficile* strains. In the quoted research, HRM results of the multiplex real-time PCR amplification products of the *tcdB*, *tcdC* and *cdtB* genes were confirmed by microchip electrophoresis. However, only three strains were subjected to such analysis. However, it has been proven that two hypervirulent strains can be effectively distinguished from a non-hypervirulent counterparts. Additionally, using electrophoretic separation of the amplified DNA, strains belonging to 027 and 078 ribotypes were also successfully distinguished.

In the review by Tamburro and Ripabelli [17], on the benefits of using the HRM method in molecular microbiology, the use of multiplex PCR combined with HRM for the identification of *C. difficile* ribotypes 027 and 078 was mentioned. The same review also demonstrated the use of the mentioned method to be quick and cost-effective in identifying rifaximin-resistant *C. difficile* strains and for determining of subtypes resulting from the occurrence of single-nucleotide polymorphisms in their DNA [17].

The combination of PCR and HRM has previously been used to detect and identify individual bacteria, including strains of the *C. difficile* species, by Bender et al. [18]. In the cited experiment, the used primers amplified a fragment of the *tcdA* gene, the Tm of which was 80.37 ± 0.45 °C. The use of this method showed that Tm values are unique for the given species DNA fragments. Moreover, the obtained values were repeatable and reproducible, and the primers enabled precise amplification, which was also confirmed by gel electrophoresis of amplicons.

The usefulness of the method, based on real-time HRM-PCR in molecular microbiology diagnostics of gastrointestinal infections, was also demonstrated in the study by Singh et al. [19] focusing on the detection of *Escherichia coli* O26 and O111 serotypes with Shiga toxin genes. In the experiment, the primers used for both serogroups showed 100% specificity. Potentially virulent strains produced distinct melting profiles, focusing separately on melting point plots. Thanks to this, they could be easily distinguished from non-virulent strains. Analysis of serotype O26 showed 75% specificity and 100% sensitivity, while analysis of serotype O111 showed 100% specificity and 100% sensitivity in identifying potentially virulent strains.

Nowadays, an important aspect may be the ongoing discussion regarding variability in antibiotic resistance profiles among binary toxin-producing *C. difficile* isolates. The study by McDonald et al. [20] demonstrated a higher occurrence of fluoroquinolone resistance in *C. difficile* strains, with variations observed in toxin genes. As noted by Androga et al. [21] *C. difficile* A^−^B^−^CDT^+^ strains can induce additional antibiotic resistance mechanisms, enhancing their environmental adaptability, and thereby increasing their ability to survive and effectively colonize the host macroorganism. Reduced antibiotic susceptibility is not directly associated with severe or recurrent *C. difficile* infection. However, *C. difficile* strains with the binary toxin gene in general show lower sensitivity levels to antimicrobial agents. An important aspect is also the geographic distribution of strains. Studies from Korea have shown susceptibility to clindamycin and moxifloxacin in 68.9% and 62.1% of cases, respectively [22]. On the other hand, studies by Costa et al. [23] demonstrated that the antimicrobial susceptibility of *C. difficile* is reduced in strains positive for the binary toxin gene. This study may serve as a preliminary step for further research on the correlation between the presence of antibiotic-resistant genes among CDT-positive strains.

In turn, the HRM-PCR test designed by Pakbin et al. [24] to distinguish four *Shigella* species was targeted at the *rrsA* gene. They analyzed 49 samples, both of clinical and food origin. The assay demonstrated relatively high analytical sensitivity in the presence of only 0.01–0.1 ng of input template DNA and analytical specificity of 100% for the differentiation of *Shigella* species. The PCR-HRM test also enabled the correct identification of the species for all tested *Shigella* isolates. The use of the unique Tm value of amplification products was also used by Reese and Elkins [25] to develop a test to detect and identify separately and simultaneously three species of bacteria causing foodborne epidemics: *Campylobacter jejuni*, *E. coli* and *Salmonella enterica*. The used primers were finding the specific and unique gene sequence of each pathogen, including: *cadF*, *yedN* and *hilA*, respectively. Each species was identified by its characteristic Tm in a single test: 78.10 ± 0.58 °C for *C. jejuni*, 81.96 ± 0.42 °C for *E. coli*, and 87.55 ± 0.37 °C for *S. enterica*. Using the multiplex variant, all the three pathogens were successfully detected and identified with clearly separated peaks. The determinations mentioned were performed five times on different days, and the results obtained were repeatable.

In the current study, the high-resolution melting PCR test has also been proven to be specific, rapid, and highly sensitive in experiments. The occurrence of small differences in Tm values obtained for the amplification products of individual genes is most likely due to the detection of different *C. difficile* genotypes with their DNA polymorphisms. The presence of single-nucleotide changes may already cause such a change [26]. Differences in the Tm values obtained for the same genes detected with different thermal cyclers may also result from their technical parameters. In this aspect, the quality of the research equipment itself plays a significant role, as even millisecond differences in heating change the following reading of the fluorescence intensity of the melting amplification product. The accuracy of calculating the value for the peak, which is a logarithmic reflection of the fluorescence intensity for the product melting curve, is also important. It is on this basis that the final Tm value is read.

Small differences in the light excitation and emission wavelengths in the detection channel may also contribute to the appearance of noticeable differences in the obtained Tm values of the detected genes. In the case of the CFX Opus 96 Dx Real-Time PCR System, the excitation wavelength was 470 nm, and the emission wavelength was 520 nm. In turn, for the LightCycler^®^ 480 II instrument, the measurement of the detected genes fluorescence was performed at an excitation wavelength of 465 nm and the emission wavelength at 510 nm.

Some of the diagnostic parameters obtained in our study are, at some point, relatively low, despite the undeniable potential of the established methodology. This is most likely due to the insufficient and proper optimization of the PCR reaction. It is also possible that the results obtained using the eazyplex^®^ tests, which constitute a reference point for real-time HRM-PCR results, were not 100% consistent with the actual situation, thus generating an underestimation of the values of the analyzed parameters. The sequence of the primer used to detect CDT using the eazyplex^®^ *C. difficile* complete tests (AmplexDiagnostics GmbH, Gars am Inn, Germany) is unfortunately unknown due to the manufacturer’s confidentiality and policies. The lack of this information makes it impossible to compare the nucleotide compatibility of the primers used.

The type of test material used is also an important issue for the evaluated test specificity and its overall diagnostic utility. It is common knowledge that stool samples are among the most difficult to use for molecular biology research. It would be reasonable to initially purify the material by selective DNA isolation. However, this would significantly extend the testing time and increase its costs. This was also not the intention of this study, the main goal of which was to assess the presence of *C. difficile* toxinogenic potential directly in stool samples.

Of note, the results of the analysis of samples purchased from the international quality assessment program in laboratory medicine, Labquality, were consistent and identical to those obtained in routine diagnostics. This agreement confirms the obtained results; however, the obtained moderate repeatability of our result, ranging from 64.52% to 77.42% for the detected genes, may contradict this. These values were underestimated by verifying discrepancies with eazyplex^®^ tests. This was the main reason for re-measuring of the same gene in the same sample and finally resulting in lowering the diagnostic parameters. Pipetting errors might have also contributed to this state of affairs. The negative result could have also resulted from typical human errors, e.g., mixing up the reaction mixture or adding too small a volume or even omitting one of the ingredients necessary for the reaction to occur. In turn, a positive result could have resulted from contamination of the samples or the thermal cycler, for example, caused by the inappropriate sealing of the tubes with an adhesive foil.

The detection of only one gene encoding one of the CDT subunits in four samples is not synonymous with the result of FN or FP. Due to the repeated testing, the chances of such a situation occurring were minimized, but it is not possible to completely eliminate such a risk. The most likely scenario is the occurrence of mutations, e.g., point mutations, within the gene sequence to which the primer is attached, preventing efficient and repeatable amplification. This assumption could been confirmed by, for example, sequencing the DNA obtained from a strain growing from such a sample.

Nevertheless, the application of the established proposed innovative diagnostic method enables fast and easy detection of genes encoding CDT subunits. However, given the potential of this pioneering method, further research is required to optimize it or even expand it to detect other *C. difficile* toxins genes.

## 5. Conclusions

Real-time HRM-PCR is a relatively simple molecular biology-based method enabling the rapid detection of *C. difficile* genes directly in stool samples. The use of real-time HRM-PCR for the detection of CDT subunit genes is characterized by high specificity and NPV and moderate sensitivity along with PPV. In order to standardize the interpretation criteria, measurements should always be performed in one type of thermal cycler. The diagnostic evaluation parameters of the real-time HRM-PCR technique demonstrate significant potential for the usefulness of this method as a standard microbiological procedure for the identification of hypervirulent *C. difficile* strains; however, the method still requires optimization or expansion to other toxin genes.

## Figures and Tables

**Table 1:** The specification of the primers used in the study.

Name	Gene	Primer Sequence 5′→3′	Tm [°C]	Amplicon Size [bp]
cdtA-FA		-GGGAAGCACTATATTAAAGCAGAAGC-	64.8	221
cdtA-FB	*cdtA*	-GGGAAACATTATATTAAAGCAGAAGC-	62.7	
cdtA-R		-CTGGGTTAGGATTATTTACTGGACCA-	65.6	
cdtB-F	*cdtB*	-TTGACCCAAAGTTGATGTCTGATTG-	67.8	262
cdtB-R		-CGGATCTCTTGCTTCAGTCTTTATAG-	64.1	
GluD-F	*gluD*	-GTCTTGGATGGTTGATGAGTAC-	59.9	158
GluD-R		-TTCCTAATTTAGCAGCAGCTTC-	61.4	

bp—base pairs; *cdtA*—binary toxin subunit A gene; *cdtB*—binary toxin subunit B gene; *gluD*—glutamate dehydrogenase gene; Tm—melting temperature.

**Table 2:** The detailed list of concentrations and volumes of reagents for preparing the reaction mixture used in this study.

Reagent	Initial Concentration	Volume of Reagent in the Reaction Mixture [µL]
5× HOT FIREPol^®^ EvaGreen^®^ HRM Mix (SolisBiodyne)	5×	4
Primer F for *cdtB* and *gluD* or Primers FA and FB for *cdtA* (50-fold diluted)	100 µM	2.5 or 2 × 1.25
Primer R (50-fold diluted)	100 µM	2.5
PCR-grade water	-	8
DNA	-	3
Total	-	20

**Table 3:** Melting temperature values of the detected genes fragments.

	Tm Values of the Detected Genes Fragments [°C]		
Analyzer	*gluD*	*cdtA*	*cdtB*
CFX Opus 96 Dx Real-Time PCR System	80.5–81	77.5–78.5	78.5–80
LightCycler^®^ 480 II	83.5–84	80–81	- *

*cdtA*—binary toxin subunit A gene; *cdtB*—binary toxin subunit B gene; *gluD*—glutamate dehydrogenase gene; Tm—melting temperature peak; *—the *cdtB* gene was detected using the CFX Opus 96 Dx Real-Time PCR System analyzer only.

**Table 4:** Comparison of the number of the detected genes and the level of statistical significance in the analysis of differences in the frequency of detected *C. difficile* genes in clinical samples (*n* = 276).

Gene	*cdtA*	*cdtB*	*gluD*	*p* Value
presence	15	17	189	0.0277
absence	261	259	87	

*cdtA*—binary toxin subunit A gene; *cdtB*—binary toxin subunit B gene; *gluD*—glutamate dehydrogenase gene; *n*—number of the obtained results; *p* value—probability value.

**Table 5:** Comparison of the results obtained by real-time HRM-PCR with the comparative, IVD approved method, eazyplex^®^ test results (*n* = 278).

Result	Gene		
	*gluD*	*cdtA*	*cdtB*
TP	156	10	10
TN	84	258	258
FP	35	6	7
FN	3	4	3

*cdtA*—binary toxin subunit A gene; *cdtB*—binary toxin subunit B gene; FN—number of false-negative results; FP—number of false-positive results; *gluD*—glutamate dehydrogenase gene; TN—number of true-negative results; TP—number of true-positive results.

**Table 6:** Assessment of diagnostic parameters of the performed tests.

Parameter	Gene		
	*gluD*	*cdtA*	*cdtB*
sensitivity [%]	98.11	71.43	76.92
specificity [%]	70.59	97.73	97.36
PPV [%]	81.68	62.50	58.82
NPV [%]	96.55	98.47	98.85

*cdtA*—binary toxin subunit A gene; *cdtB*—binary toxin subunit B gene; *gluD*—glutamate dehydrogenase gene; NPV—negative predictive value; PPV—positive predictive value.

**Table 7:** Assessment of the repeatability of the determinations performed for a selected samples with a corresponding number, mentioned below; each testing for repeatability was conducted twice for the same sample.

	Gene		
	*gluD*	*cdtA*	*cdtB*
*n*	62	31	35
Compatibility of the results	40	24	25
Repeatability [%]	64.52	77.42	71.43

*cdtA*—binary toxin subunit A gene; *cdtB*—binary toxin subunit B gene; *gluD*—glutamate dehydrogenase gene; *n*—number of the obtained results.

## Data Availability

The raw data supporting the conclusions of this article will be made available by the authors on request.
